# Binding Free Energy Landscape of Domain-Peptide Interactions

**DOI:** 10.1371/journal.pcbi.1002131

**Published:** 2011-08-18

**Authors:** Iskra Staneva, Stefan Wallin

**Affiliations:** Department of Astronomy and Theoretical Physics, Computational Biology and Biological Physics Group, Lund University, Lund, Sweden; UNC Charlotte, United States of America

## Abstract

Peptide recognition domains (PRDs) are ubiquitous protein domains which mediate large numbers of protein interactions in the cell. How these PRDs are able to recognize peptide sequences in a rapid and specific manner is incompletely understood. We explore the peptide binding process of PDZ domains, a large PRD family, from an equilibrium perspective using an all-atom Monte Carlo (MC) approach. Our focus is two different PDZ domains representing two major PDZ classes, I and II. For both domains, a binding free energy surface with a strong bias toward the native bound state is found. Moreover, both domains exhibit a binding process in which the peptides are mostly either bound at the PDZ binding pocket or else interact little with the domain surface. Consistent with this, various binding observables show a temperature dependence well described by a simple two-state model. We also find important differences in the details between the two domains. While both domains exhibit well-defined binding free energy barriers, the class I barrier is significantly weaker than the one for class II. To probe this issue further, we apply our method to a PDZ domain with dual specificity for class I and II peptides, and find an analogous difference in their binding free energy barriers. Lastly, we perform a large number of fixed-temperature MC kinetics trajectories under binding conditions. These trajectories reveal significantly slower binding dynamics for the class II domain relative to class I. Our combined results are consistent with a binding mechanism in which the peptide C terminal residue binds in an initial, rate-limiting step.

## Author Summary

The complex biological processes occurring in living organisms are enabled by numerous networks of interacting proteins. It is therefore of great interest to understand the physical interplay between proteins and, in particular, how this process gives rise to highly specific network connectivities. For a long time, the dominant molecular view of protein-protein interactions was the docking of more or less static folded structures, with specificity obtained from a complementarity in shape and charge distributions. Lately it has been realized that many of the links in protein networks are mediated by interactions between folded domains, on the one hand, and disordered polypeptide segments, on the other. We use an all-atom Monte Carlo based approach which attempts to capture this domain-peptide binding process in full and apply it to representative members of a common domain family. This allows us to examine and compare detailed aspects of the binding free energy landscapes which underlie specificity and affinity. Being able to model domain-peptide binding in a physically sound, yet computationally tractable way is essential for identifying molecular binding mechanisms and opens up possibilities for modifying interaction networks in a controlled way.

## Introduction

Protein-protein interactions control numerous processes in the cell. Recently, it has been realized that a significant fraction of these interactions are mediated by the binding of flexible polypeptide segments to folded domains [1]–[3]. This realization is in part due to the discovery of many so-called peptide recognition domains (PRDs), which function specifically by recognizing sets of short peptide sequences [4], [5]. PRDs often interact with their ligand peptides in a reversible, transient manner, making them particularly well suited to mediate interactions in signaling and regulatory processes, which require fast response to initiated or ceased stimuli. A fundamental understanding of the detailed dynamics and binding free energy landscapes of these PRD-peptide interactions will therefore eventually be necessary in order to understand the finely tuned specificities and affinities which underpin many protein interaction networks. Achieving such an understanding may also be of practical importance, as it can be a starting point towards altering signaling networks in a controlled way [6], [7] or designing small molecules to inhibit domain-peptide binding [8], [9].

Modeling peptide binding in atomistic detail is a challenge. One reason for this is the inherent flexibility of a disordered peptide chain which necessitates a statistical mechanical approach. At the same time it is a major modeling opportunity because of the relatively small molecular interface and few amino acids involved, making the peptide binding process computationally accessible. Several docking methods designed specifically for peptide binding have been developed [10]–[16], which aim to predict the correct peptide binding pose on a protein surface. Most of these methods require some prior knowledge of the peptide binding site, although true blind docking has also been attempted [17], [18]. Other *in silico* methods seek to provide binding predictions for whole PRD families, including SH2 [19], SH3 [20], [21], and PDZ [22] domains. These methods rely on structural models of domain-peptide complexes using an available experimental peptide-bound configuration as a template. Most PRD families, however, display significant diversity in how peptides interact with domains, which fundamentally limits this approach. In a recent effort to alleviate this problem, King *et al* [15] combined peptide docking and subsequent structure-based binding prediction using the Rosetta scoring function. Molecular Dynamics simulations of domain-peptide bound states have also been carried out, emphasizing the importance of dynamics and flexibility for understanding the molecular basis of peptide binding [23]–[25].

Our aim here is to go beyond docking and investigate the binding process from an equilibrium perspective. To this end, we use a recently developed Monte Carlo-based procedure for protein-peptide binding [26] and apply it to three different PDZ domains and their target peptide sequences. The approach combines a global conformational search of the peptide chain, as well as limited protein backbone flexibility around the native state, with an effective energy function inspired by protein folding studies [27]–[29]. Rather than relying on large numbers of docking attempts, we perform fewer but long simulations such that each run exhibits multiple binding and unbinding events, thereby providing an equilibrium picture of the binding process. In particular, this allows us to investigate and compare features of the global binding free energy landscape as determined by the interaction between the protein surface and the amino acid sequence of the peptide.

The PDZ domain is an archetypical PRD existing in large numbers in many genomes [30]–[32]. It distinguishes itself from other PRDs in that it typically binds sequence motifs at the extreme C terminal end of proteins. The architecture is mostly conserved across the domain family with a typical core structure consisting of two 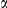-helices and six 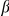-strands. The PDZ fold includes a binding pocket between the second 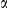-helix (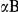) and second 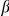-strand (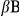) such that a ligand peptide can augment the 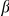-strand upon binding and pack its sidechains against the 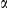-helix. In addition, the peptide C terminus forms hydrogen bonds with the backbone amides of a highly conserved loop on the PDZ domain. Like many other PRD families, PDZ domains have been divided into different classes depending on which peptide sequences they preferentially bind. The most established division of PDZ domains is into classes I, II, and III, corresponding to the sequence patterns Ser/Thr-X-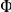-COOH, 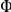-X-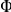-COOH, and Asp/Glu-X-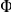-COOH, respectively, where 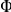 is any hydrophobic amino acid, X is any amino acid, and COOH is the C terminus [32]. It can be pointed out that more fine-grained classifications are also possible [33]. We focus here on comparing the binding behavior of class I and II domains, which represent the majority of known PDZ domains [30], [32].

An important aspect of any binding study is the ability to capture binding to free molecules, i.e., to structures determined in the absence of a ligand. This is important not the least for PDZ domains, for which only 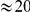 domain-peptide complexes have been solved experimentally so far [32], compared to the almost 200 free PDZ domain structures in the Protein Data Bank (PDB) [34]. We therefore start out by testing our computational procedure using two different structural forms of the domains, free and peptide-bound. Thereafter, we describe the conformational transitions of the peptides and the binding free energy landscapes for the domains. Finally, we perform a large number of Monte Carlo based kinetic simulations to obtain a deeper microscopic picture of the peptide binding process.

## Results/Discussion

### Selected Protein Domains

As class I and class II representatives we chose the 3rd PDZ domain of PSD-95 and the 6th PDZ domain of GRIP1, respectively. These are typical class I and II PDZ domains in the sense that all known binding peptides fall within their respective ideal class motifs [30], [35]. Free and peptide-bound X-ray structures have been determined for both domains [36], [37], and for PSD-95 the binding thermodynamics [38] as well as kinetics [39], [40] have been particularly well characterized. The ligands present in the two peptide-bound structures were derived from the C termini of the proteins CRIPT (PSD-95) [36] and human Liprin-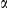 (GRIP1) [37]. We consider here the binding of these two ligands to both the bound (b) and free (f) structural forms and denote the systems by PSD95-Ib, PSD95-If, GRIP1-IIb, and GRIP1-IIf, respectively. In addition to these class I and II domains, we include in this study the PDZ domain of PICK1 which is one of the few known PDZ domains with dual class I and II specificity. The structure of PICK1 PDZ has been determined with class II peptides [41], [42]. We consider binding of ligands taken from protein kinase 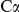 (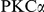, class I) and AMPA receptor subunit GluR2 (GluR2, class II), which are known binders to PICK1 [43], [44], and denote the systems with PICK1-Ib and PICK1-IIb, respectively. The PDZ domains and peptide amino acid sequences under study are summarized in Table 1.

**Table 1:** PDZ domains and peptide sequences studied.

Abbreviation	PDB ID	Exp	Peptide sequence	Peptide name
PSD95-Ib/PSD95-If	1BE9/1BFE	X-ray	KQTSV	CRIPT
GRIP1-IIb/GRIP1-IIf	1N7F/1N7E	X-ray	ATVRTYSC	Liprin- 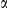
PICK1-Ib	–-	–-	LQSAV	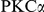
PICK1-IIb	2PKU	NMR	ESVKI	GluR2

### Simulation Procedure and Minimum-Energy Conformations

To simulate the domain-peptide binding process, we use the MC based approach developed in Ref. [26]. This simulation procedure is general in that it can in principle be applied to any protein-peptide pair as long as a protein structure is available. Briefly, it works in the following way. A relaxed protein domain structure is centered in a cubic box and joined by a peptide in a random conformation away from the protein surface. The peptide is entirely free to search conformational space, restricted only by periodic boundary conditions on the box. The protein, on the other hand, is kept close to its native structure using constraints on the 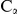-atoms, which allow limited backbone and in principle full sidechain flexibility. We combine this simple procedure with an implicit-solvent all-atom energy function based on effective hydrogen bond, electrostatic, and hydrophobic forces [26]. Here we improve the model by including a context-dependent desolvation effect for backbone atoms groups (see Methods). We find, in particular, that including such a context-dependence improves the challenging case of simulating peptide binding to free domain structures. Energies *E* and temperatures *T* are given in dimensionless model units.

The thermodynamic behavior of our systems is obtained using Simulated Tempering (ST) [45]–[47], an expanded ensemble MC method in which *T* is treated as a dynamical parameter. The method is convenient both for finding global minimum-energy states and studying equilibrium behavior. For each PSD-95 and GRIP1 structure-peptide pair, we performed 5 independent ST runs. An example trajectory is shown in Figure S1 in Supporting Information. In addition, fixed-*T* MC simulations close to the midpoint, 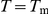, i.e., where bound and unbound populations are equal, were also performed to provide additional statistics for free energy surface calculations. 10 independent fixed-*T* runs were performed for each structure-peptide pair in Table 1. Additional details on the computational model and simulation procedure are provided in Methods.

**Figure 1:**
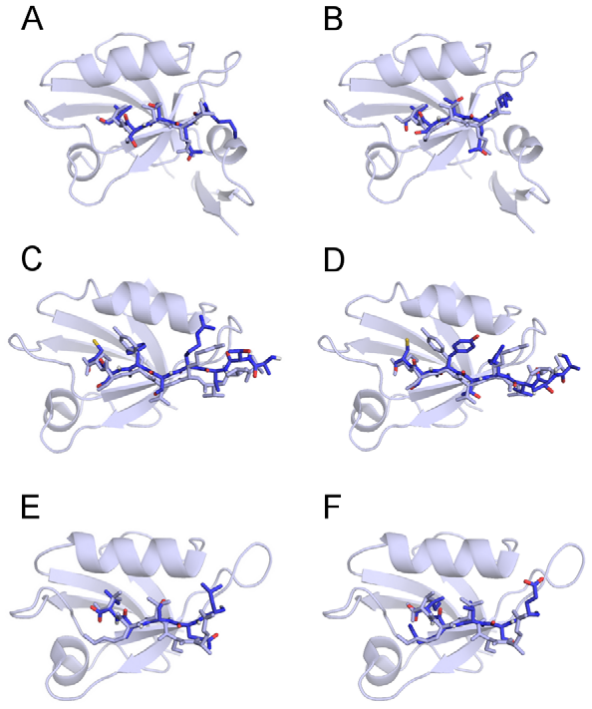
Minimum-energy peptide conformations found across all simulations for (A) PSD95-Ib, (B) PSD95-If, (C) GRIP1-IIb, (D) GRIP1-IIf, (E) PICK1-Ib, and (F) PICK1-IIb. Nitrogen and carbon are shown in dark blue, oxygen in red, sulfur in yellow, and hydrogen in white. Experimentally determined domain-peptide complexes with PDB IDs (A, B) 1BE9, (B, C) 1N7F, and (D, E) 2PKU are shown in uniform light blue. The corresponding 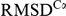 values between model and experimental peptide conformations are 0.9, 1.1, 1.7, 1.7, 2.4, and 2.3 Å, respectively (see Equation 1).

A challenging test for our computational model, used also in guiding the development of our all-atom energy function, is the prediction of bound peptide conformations. Figure 1 shows the model conformations found with the lowest total energy, *E*, across all ST and fixed-*T* MC runs for each system, superimposed on the corresponding experimental structures. All 6 min-*E* conformations are bound at the PDZ peptide binding pocket and many of the finer atom-level details match the experimental structures. Of special interest is to compare the two sets of results obtained for the ligand-bound and ligand-free PSD-95 and GRIP1 PDZ domain structures. One of the most pronounced differences is due to the different sidechain orientations at P(–2) between GRIP1-IIb and GRIP1-IIf docked peptides, such that the Tyr sidechain is pointing either out (GRIP1-IIf) or into (GRIP1-IIb) the peptide binding pocket (residue positions on PDZ binding peptides are typically numbered P(0) for the C terminus residue, P(–1) for the immediately preceding residue, and so on). This difference in orientation is likely related to a small shift in the 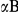 helix between the ligand-free and ligand-bound structures of the GRIP1 domain [37], such that the binding pocket is slightly wider in the bound structure.

### Free vs Peptide-Bound Domain Structures

**Figure 2:**
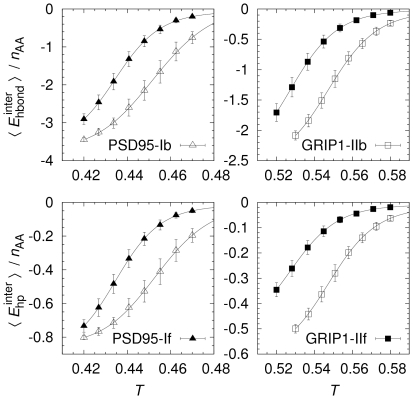
Equilibrium peptide binding curves. Thermodynamic averages of inter-chain hydrogen bond (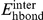) and hydrophobicity (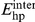) energies as a function of temperature, *T*, normalized by the number of peptide amino acids, 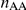 (we note that the expression for the hydrophobicity energy, 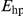, equation 4 in Ref. [26], contains an overall sign error which we correct here; in all calculations, 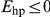, and consequently 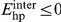, as it should be). Solid lines are fits to a simple model assuming only two states, bound (B) and unbound (U), in which the temperature dependence of an observable *X* has the functional form 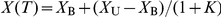, where 
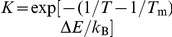
, 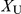 and 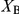 are observable baseline values, 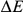 is the energy difference between U and B, and 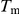 is the midpoint temperature. All statistical errors in this and other plots are jackknife estimates indicating 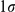 errors.

Having seen that the lowest-*E* states represent more or less correctly bound ligands, we turn to the equilibrium behavior of the domain-peptide interaction. Figure 2 shows the *T* dependence of inter-chain hydrogen bond and hydrophobic interactions for PSD95-If/b and GRIP1-IIf/b. Some general trends are immediately seen. At high *T*s, only limited interactions between peptides and domains occur, consistent with a process dominated by entropic effects. As *T* is lowered, peptides and domains associate increasingly, making both favorable hydrogen bonds and hydrophobic interactions. While all binding curves are smooth, the precise behavior is seen to depend on which domain structure type is used. Particularly, we find that the free domain structures (PSD95-If and GRIP1-IIf) bind their ligands somewhat weaker than their respective bound structures (PSD95-Ib and GRIP1-IIb).

**Figure 3:**
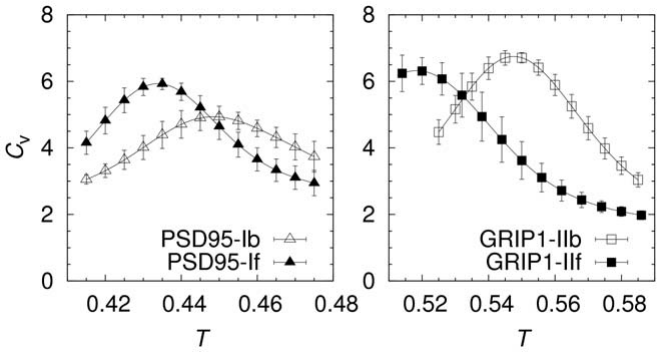
Specific heat capacity as a function of temperature. The specific heat is calculated using 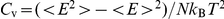, where *N* is the total number of amino acids, *E* is the total energy, and 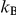 is the Boltzmann constant (taken to be 1 in this work).

To investigate this difference quantitatively, we fit the binding curves in Figure 2 to a simple two-state expression with 4 free parameters. The fits are good for all binding curves and the fitted parameters are given in Tables 2 and 3. Of particular interest are the parameters 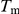, the midpoint temperature representing equal populations of the two states, and 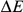, the energy difference which controls the sharpness of the transition. The midpoints obtained are 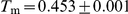 and 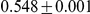 for PSD95-Ib and GRIP1-IIb, respectively. The corresponding 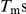 for PSD95-If and GRIP1-IIf are roughly 4% lower. We also find differences in 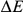, as well as in the other 2 fit parameters, but the statistical errors for these parameters are larger (see Table 2 and 3). One statistically significant difference is a slightly sharper binding transition for PSD95-If compared to PSD95-Ib. This can also be seen as a relatively higher peak in the specific heat capacity curve (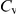) for PSD95-If, as shown in Figure 3. However, all 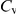 curves exhibit single peak behavior and the *T*-values at the 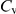 peaks correspond well to the 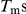 found from the fits in Figure 2. Hence, while we find differences in the binding behavior for bound and free domain structures, binding as an overall two-state process with a single transition appears to be a robust feature.

**Table 2:** Parameter values for two-state fits to ligand binding curves for PSD95-Ib and PSD95-If.

Parameter	PSD95-Ib/ 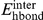	PSD95-Ib/ 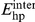	PSD95-If/ 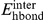	PSD95-If/ 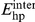
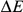	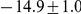	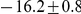	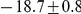	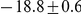
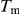	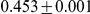	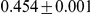	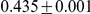	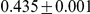
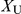	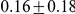	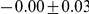	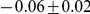	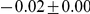
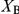	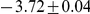	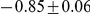	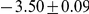	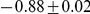

**Table 3:** Parameter values for two-state fits to ligand binding curves for GRIP1-IIb and GRIP1-IIf.

Parameter	GRIP1-IIb/ 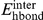	GRIP1-IIb/ 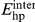	GRIP1-IIf/ 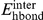	GRIP1-IIf/ 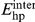
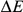	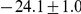	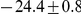	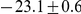	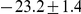
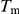	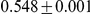	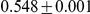	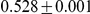	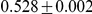
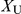	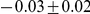	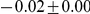	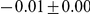	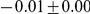
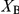	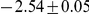	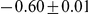	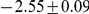	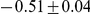

The variations in binding behavior between bound and free structures obtained in our simulations reflect structural differences between liganded and unliganded PDZ domain forms. Some of these differences are likely preserved by our native state constraints. Previous simulation results indicate that overall receptor flexibility and dynamics can play a major role in PDZ peptide binding and selectivity [7], [25], [48], [49]. Interestingly, structural differences in the binding pocket between bound and free form is significant for the GRIP1 domain [37] while quite negligible for PSD-95 [36]. Our results thus indicate that even subtle structural differences can impact binding significantly. Regardless of these differences between bound and free form our model predicts that the GRIP1 domain binds its peptide more strongly than PSD-95, with 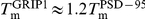 (see Figure 2). Meaningful quantitative binding affinities cannot be directly obtained, however, because *T* is not matched to physical units. Experimentally, the dissociation constant of the PSD-95/CRIPT interaction has been measured to 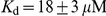 at 298 Kelvin, using isothermal titration calorimetry [38]. The binding affinity of the GRIP1 domain for the Liprin-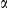 peptide has to our knowledge not yet been determined.

### Binding vs Folding

The binding curves in Figure 2 report on the overall character of the binding transition but do not provide any structural details, such as where on the protein surface binding preferentially occurs or how the peptide chain dynamics is influenced by binding. In defining a bound state, we use the root-mean-square-deviation between the atom coordinates of a model peptide conformation, 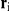, and those of the experimental (native) peptide structure, 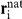, i.e.,
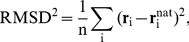
(1)where the sum goes over n peptide atoms, either all non-H or only 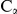-atoms (indicated by superscripts ALL and 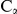, respectively). An advantage of the RMSD measure is that a small value indicates that binding has occurred both at the right surface area and with a native-like internal conformation. Any peptide with 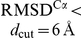 is considered correctly bound in the PDZ binding pocket. The choice of 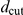 will be discussed later. In order to delineate the internal conformational dynamics of the peptide chain from its binding, we calculate also 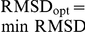, where the minimization is over all rigid body translations and rotations of the peptide conformation. Hence, 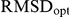 is the measure typically used in the analysis of folding trajectories and its notation is chosen merely to distinguish it from the “non-optimized” RMSD measure in Equation 1. A small 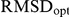 means that the peptide is native-like regardless of whether it is bound or not.

**Figure 4:**
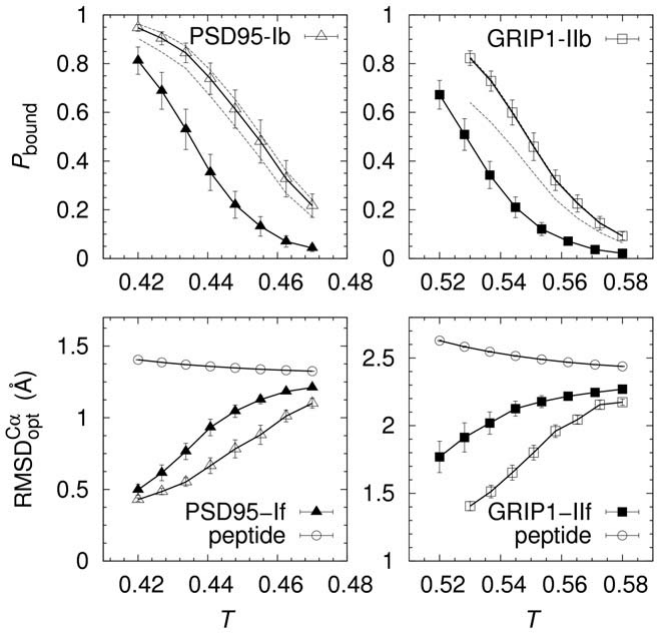
Domain-peptide binding as a minimal example of coupled folding and binding. The probability for a peptide chain to occupy the PDZ peptide binding pocket, 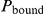, increases with decreasing temperature, *T*. The solid curves are obtained with 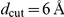. Dotted curves indicate 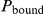 values obtained with 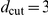 and 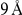, respectively, for PSD95-Ib and GRIP1-IIb. Peptide binding is mirrored by an increasing internal similarity with the corresponding native peptide structures, as manifested by a decrease in 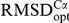. No such decrease in 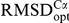 is seen for isolated peptides.

For both the PSD-95 and GRIP1 domain-peptide pairs, the probability that the peptides occupy the bound state, 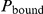, increases sharply as *T* is lowered (see Figure 4). It is notable that for PSD95-Ib, at the lowest *T* simulated, 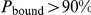, indicating a very low probability for the peptide to bind parts of the domain surface other than the PDZ binding pocket. 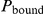 values for PSD95-If, GRIP1-IIb, and GRIP1-IIf are lower but the PDZ binding pocket is the dominating binding site in these cases, too, and 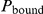 will likely increase further at still lower *T*s. Consistent with our results in Figure 2, Figure 4 shows a higher peptide binding propensity for liganded (PSD95-Ib and GRIP1-IIb) compared to the unliganded structures (PSD95-If and GRIP1-IIf). These shifts are smaller than the differences between the two PDZ domains, as noted above.

When the peptides associate with the protein surfaces they not only bind to the peptide binding pocket, they also undergo internal conformational transitions such that they more closely resemble the native peptide structures. This is clear from the lower panel of Figure 4, which shows that 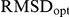 decreases with temperature *T*. Hence, the peptide-binding process also leads to increasingly native-like peptide conformations. By contrast, the peptide chains by themselves show little tendency to form any specific structure, at least over the temperatures studied, as indicated by a relative constant 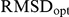 for isolated chains (see Figure 4). Moreover, the chain compactness is similarly only weakly dependent on *T* for both peptide sequences (see Figure S2 in Supporting Information). In this sense, our peptides are intrinsically disordered and their interaction with the PDZ domains can be seen as a minimal example of coupled folding and binding. Direct observation of such coupled folding-binding behavior in atomistic simulations has been seen previously mainly for 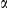-helical peptides [50]–[54].

It must be pointed out that despite the indicated “folding,” significant structural heterogeneity remains in the bound state. This diversity represents the conformational entropy of the bound state and is important to take into account since it can significantly contribute to ligand binding [55]–[57]. In fact, in defining the bound state, our aim was to choose 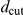 large enough to comprise most of this diversity, but not too large such that incorrectly bound peptide conformations are included. To explore this tradeoff, we show in Figure 4 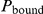 curves obtained also with 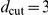 and 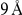 for PSD95-Ib and GRIP1-IIb. Increasing 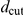 to 9 Å from 6 Å has a relatively small impact on the 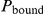 curves. Most of the structural diversity is therefore included with 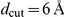. At the other end, to see that 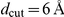 is not too large, we superimposed representative sets of peptide conformations with 
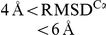
. This ensemble is naturally diverse but do not include conformations that can be considered misdocked (see Figure S3 in Supporting Information). Finally, we find it instructive to construct reference structures by rotating the experimental peptide structures by a half turn, such that the 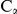 atoms of the first and last peptide amino acids exchange positions. These “flipped” peptides have 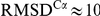 and 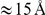 for the CRIPT (PSD-95) and Liprin-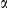 (GRIP1) peptides, respectively. Hence, peptide conformations of this nature would not contribute positively towards 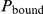 in our definition of the bound state (and are not observed in our simulations).

### Binding Free Energy Surfaces

**Figure 5:**
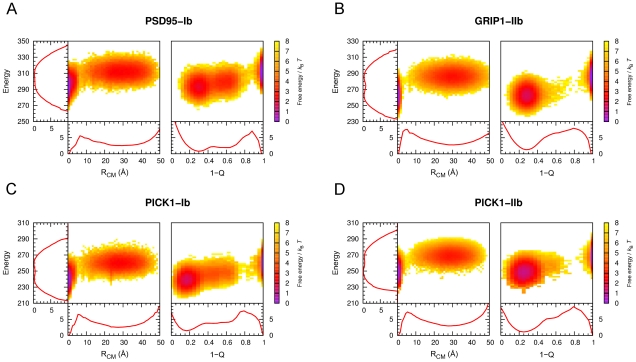
Peptide binding free energy surfaces for PSD95-Ib, GRIP1-IIb, PICK1-Ib, and PICK1-IIb, at 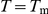
 **.** Free energies are calculated using 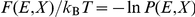, where 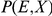 is the joint probability distribution in total energy, *E*, and 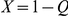 or 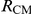. The one-dimensional free energy profiles are obtained from the corresponding marginal distributions. PSD95-Ib and GRIP1-IIb free energies were calculated directly from fixed-*T* MC simulations at the respective 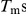. PICK1-Ib and PICK1-IIb simulations were performed at 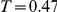 and 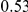, respectively. Free energies at the midpoints (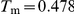 for PICK1-Ib and 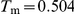 for PICK1-IIb, determined from the 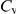 maxima) were obtained using single-histogram reweighting [64].

We turn now to the binding free energy landscapes of our PDZ domains, i.e., the free energy as a function of a set of order parameters indicating the progress of binding. For this purpose we use, in addition to the total energy *E*, two standard [58], [59] structural order parameters, 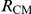 and *Q*, defined as the distance between the centers-of-mass (CM) of model and experimental peptide conformations and the fraction of inter-chain native contacts, respectively. 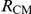 and *Q* are complementary in that each provide different perspective on the peptide binding process. The binding free energy surfaces for PSD95-Ib and GRIP1-IIb show bound and unbound states well separated with a single barrier (the transition state, TS) at 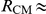
4–6 Å and 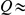
0.1–0.2 (see Figure 5). The binding landscapes do not exhibit any competing deep local minima representing misdocked conformations and therefore constitute almost ideal “binding funnels” [60]. This is reassuring in terms of the validity of the model and indicates that nonspecific binding between PDZ domain and peptide chains may be very limited.

The one-dimensional free energy profiles in 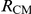, *Q* and *E* reveal a more distinct free energy barrier between the bound and unbound states for GRIP1-IIb compared to PSD95-Ib, indicating a more cooperative binding process for the class II domain (see Figure 5). In the *E* parameter, a small barrier separates bound and unbound states for GRIP1-IIb while such a barrier is mostly absent for PSD95-Ib. In the structural parameters, *Q* and 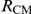, the barriers are overall much higher but the trend remains. This can be seen, for example, in the free energy difference between the transition state and the native, bound state, 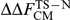, in the 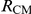 parameter. From Figure 5, we find that 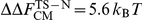 and 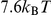 for PSD95-Ib and GRIP1-IIb, respectively. One could easily suspect that the relatively higher barrier for GRIP1-IIb is due to its longer peptide. This is however not the case. We re-made our simulations for GRIP1-IIb with a truncated, 5-amino acid version of Liprin-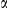 and found that 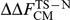 in fact increases slightly to 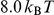. Hence, the difference between the PSD-95 and GRIP1 systems is likely mainly related to differences in the amino acid sequences. The bound state for GRIP1-IIb is characterized by a single, deep minimum at 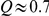, i.e., with most of the native contacts formed. The PSD-95 domain, by contrast, exhibit a significantly wider distribution of *Q*-values in the bound state. In addition to a deep 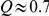 minimum, a second weaker minimum exists at 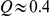. Visual inspection of the 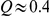 minimum reveals peptide conformations in which the C terminal Val of CRIPT is tethered to the PDZ binding pocket, kept in place mainly through hydrophobic interactions involving the Val and hydrogen bonding between the peptide C terminus and the PDZ carboxylate binding loop, leaving a floppy N terminal region. Such flexible, yet bound conformations are mostly absent for GRIP1-IIb. Instead, its peptide typically binds through both the Cys and Tyr sidechains at P(0) and P(–2). From the perspective of our model, we find that additional hydrophobic contacts provided by P(–2) in class II domain-peptide binding give a more rigidly bound peptide ensemble, which in turn produces a higher free energy barrier for binding and a more cooperative binding process.

A question that arises in comparing features of the free energy surfaces of PSD95-Ib and GRIP1-IIb is to what extent they can be controlled by the peptide sequence. In this regard, promiscuous PDZ domains which bind both class I and II peptides are of particular interest. We therefore apply our method to one such domain, the PDZ domain of PICK1, and simulate the binding of both a class I (PICK1-Ib) and a class II (PICK1-IIb) peptide, as displayed in Table 1. Despite that the two peptide sequences bind the same domain structure, their free energy surfaces are quite different (see Figure 5C and D). Specifically, the PICK1-Ib landscape exhibits striking similarities with PSD95-Ib, particularly with regard to a broad *Q*-distribution of the bound state. PICK1-IIb, on the other hand, has a binding free energy landscape similar to GRIP1-IIb, with a single well-defined native basin of attraction. The binding free energy barriers for PICK1-Ib and PICK1-IIb are 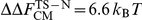 and 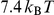, respectively, such that the class II peptide again shows a relatively stronger binding cooperativity.

It is interesting to compare our results for PICK1-Ib and PICK1-IIb with those of Madsen *et al.* [44]. Using an assay based on fluorescence polarization, they found that the PICK1 PDZ domain showed a higher affinity for a class II than a class I peptide (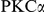). This is in qualitative agreement with our results, as we find a higher 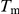 for PICK1-IIb over PICK1-Ib (see Figure 5 legend), although their class II ligand was not the same as ours. Madsen *et al.* also obtained docked peptide structures using homology modeling and found 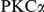 to be unusually displaced from 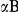 at the N terminal end, somewhat reminiscent our 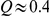 local free energy minimum. However, for typical 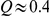 peptides in our simulations the N terminal ends have become almost entirely displaced from the 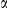-helix. One might think that this structural diversity is exaggerated by our model because, after all, PDZ specificity is in part obtained from interactions with P(–2). We therefore tested the PICK1 mutation Ala87Leu, which was introduced by Madsen *et al.* and meant to fill out the hydrophobic pocket normally occupied by the P(–2) residue. The mutation was indeed found to essentially eliminate binding to both the class I and II peptides in their assay [44]. We find in our simulations that the Ala87Leu mutation drastically reduces 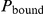 from roughly 0.5 at 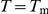 in wild-type PICK1 to 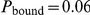 and 0.09 for the class I and II peptides, respectively. Hence, interactions involving P(–2) are still crucial for proper binding in our model despite the 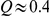 local minimum. In this context, it is interesting to note that experimental PDZ domain-peptide complexes were recently obtained in which the interaction occurs mainly through the P(0) position, such that the peptides bind roughly perpendicular to the domain surface [61].

### Monte Carlo Binding Kinetics

Above we have shown that, in our model, peptide binding can be seen roughly as a two-state process in which a single free energy barrier separates the bound and unbound states. How is this free energy barrier crossed during binding? To address this question and further investigate the mechanism underlying peptide binding we perform a large number of fixed-temperature simulations where the peptide chains are, as previously, initiated in random positions and conformations. In contrast to above, the MC “kinetics” simulations are performed using only small-step updates for the peptide chain; global, unphysical pivot moves are excluded (see Methods). A fraction of rigid body translation and rotation MC moves for the peptide chain is included. There are two processes for the peptide chain in these simulations, a search on the protein surface for the peptide-binding pocket and, subsequently, a conformational search for the correctly bound structure. Because of the inclusion of rigid body moves, we assume a dynamics in which the search process across the protein surface is fast. Relaxation towards equilibrium is therefore limited by a conformational reorganization of the peptide and protein chains during binding, which is the process we are primarily interested in.

**Figure 6:**
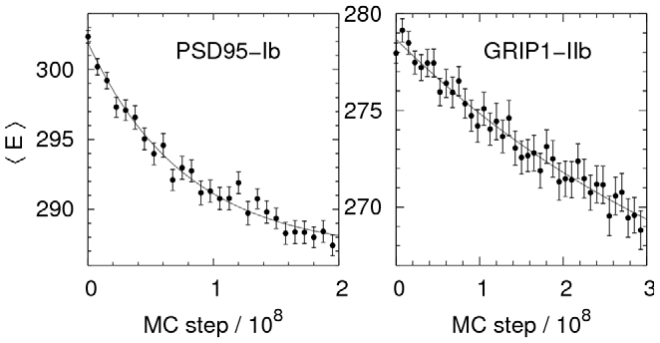
Binding relaxation curves for PSD95-Ib and GRIP1-IIb. Peptide chains are initiated in random conformations and positions away from the domain surface and thereafter evolved using fixed-*T*, small-step MC dynamics at 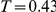 and 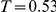 for PSD95-Ib and GRIP1-IIb, respectively. The chosen *T*s are slightly below the respective 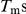. Averages are obtained from 200 independent runs. Solid lines are fits to a single-exponential curve, 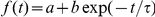, where *T* is the number of MC steps, and *a*, *b* and 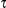 are fit parameters.

We find that the relaxation behavior for both PSD95-Ib and GRIP1-IIb systems is consistent with a single-exponential curve, as can be seen in Figure 6. This indicates a single rate-limiting step in the peptide binding process, or, in other words, the free energy barrier is crossed without significantly populating an intermediate state. Only a handful kinetic experiments of PDZ domain-peptide binding have been performed so far but one such study has presented results for the PSD-95 system analyzed here. Using stopped-flow fluorescence spectroscopy, Jemth *et al.* [39] observed single-exponential binding traces for the PSD-95 PDZ domain and a dansylated CRIPT peptide. Our results are therefore consistent with these observations. However, it must be pointed out that the MC-based simulations performed here should not be seen as mimicking kinetic experiments, as chain diffusion effects are not rigorously taken into account. A more realistic comparison is likely achieved by focusing on relative kinetic effects between peptide binding systems. In this respect, we observe a significant difference in relaxation times 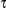 between PSD95-Ib and GRIP1-IIb, such that 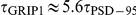, a prediction which may be tested experimentally. This difference in relaxation rate between the two domains is consistent with the larger free energy barrier seen for GRIP1-IIb over PSD95-Ib.

### Conclusions

We have developed a MC based procedure for exploring peptide binding processes and employed it to two typical PDZ class I and II domains and a dual class I–II domain. In combining the equilibrium and small-step, fixed-temperature kinetic simulation results, a picture emerges for the binding process in which there are overall similarities but also differences in the details. In all cases, binding is coupled to folding, and can be characterized as an overall two-state process with a free energy surface funneled towards the peptide bound state. Binding to the PSD-95 PDZ domain involves a lower free energy barrier than the GRIP1 PDZ domain, leading to significantly faster binding kinetics, at least for the peptide sequences studied. What is the origin of this difference? The shape of the near-native free energy surface for the GRIP1 PDZ domain indicates a relatively coherent ensemble of bound peptide conformations, stabilized by hydrophobic interactions with P(0) and P(–2). As a class I domain, the PSD-95 domain lacks strong hydrophobic interactions at P(–2) leading to a more conformationally diverse bound state, spanning a wider range of 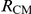 and *Q* values. In particular, we find a weak free energy minimum corresponding to peptides bound to the PDZ binding pocket mainly through the P(0) position, with a flexible N terminal tail. The population of such conformations are significantly smaller for the GRIP1 PDZ domain. Our results are therefore consistent with a binding mechanism in which the rate-limiting step is the initial binding of P(0) at the PDZ peptide binding pocket. This interpretation is also supported by recent experimental PDZ domain-peptide structures, including GRASP [61] and X11 [62], where peptides are attached in a “perpendicular” mode. To what extent these results apply to other class I and II PDZ domains remains to be seen. However, the fact that an analogous behavior is found for the dual class I–II PICK1 domain indicates that it may have some generality.

## Methods

### All-Atom Computational Model

All simulations are performed using essentially the model described in [26], with a small improvement described in the following. Our original starting point was a model developed for peptide folding [27], [28] which combines an all-atom protein representation with an effective energy function based mainly on hydrogen bonding, hydrophobicity, and electrostatic attractions. This model was then adapted for peptide binding [26], where, in particular, we added a context dependence to the energy function such that electrostatic attractions between partial charges buried in the protein were made effectively stronger than those solvent exposed. This was accomplished by using a parameter, 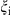, indicating the “degree of buriedness” for any atom i. In this work, we add a context-dependent term describing desolvation effects on backbone atom groups,
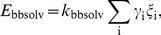
(2)in which the sum goes over all backbone NH and CO groups i. For “unsatisfied” NH and CO groups, i.e., those not participating in any intra- or inter-chain hydrogen bond, 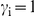, and for all others, 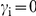. The quantity 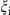 is calculated at a point, 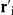, which for a NH group is located 2.0 Å from the H atom in the NH direction, and for a CO group, 2.0 Å from the O atom in the CO direction. 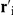 is thus found approximately in the space occupied by a potential solvent molecule hydrogen bonded to i. 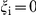 indicates that this space is available to a solvent molecule while 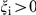 indicates it is instead occupied by other protein atoms. Hence, “unsatisfied” NH and CO groups with 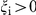 (i.e. also unlikely to participate in solvent hydrogen bonding) are energetically penalized. The term therefore acts as a desolvation effect for backbone atoms. The strength chosen is 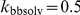. Including this energy term yields a crucially improved performance over the previous model [26], most notably for peptide binding to free domain structures. Specifically, the PSD95-If domain-peptide pair exhibited almost no propensity for correct binding previously [26] while including 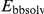 yields reliable binding as detailed in this work.

### Monte Carlo Simulations

To obtain equilibrium conformational ensembles of our domain-peptide systems we used Simulated Tempering (ST). [45]–[47], in which conformational updates are alternated with updates in the temperature *T*. Initially, a set of discrete temperatures are selected. Changes between these discrete temperatures during simulations are then treated as ordinary MC updates. 8 different temperatures in suitable ranges are used for all domain-peptide systems. For updates in conformational space, we use a few different move types. For the protein domain, which is constrained close to its native structure, we use sidechain rotations, in which a single 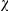-angle is turned, and semi-local backbone moves, in which 8 consecutive 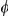
- and 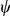-angles are turned in a coordinated way [63]. For the peptide chain, 3 additional moves are used: a pivot move which turns a single 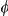
- or 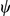-angle, and rigid body translation (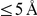) and rotation (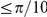) moves. An effective peptide concentration is set by the box side *L*. For computational reasons, we use a small box such that 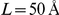, corresponding to an effective concentration of 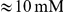.

We performed the following peptide binding simulations. For PSD95-Ib, PSD95-If, GRIP1-IIb, and GRIP1-IIf, 5 ST runs were performed with at least 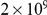 elementary MC steps. These runs were used to find the *T* dependence of various observables including the specific heat curves. 10 fixed-*T* MC runs at 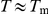 were performed for all of the 6 domain structure-peptide pairs in Table 1, each with 2 or 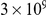 steps. These simulations were used for free energy surfaces calculations. The MC kinetic simulations differs from the equilibrium runs in the following ways. First, the global, unphysical pivot move was turned off, such that only small-step chain moves were allowed. Second, the translation step size was decreased from 5 Å to 1 Å. 200 independent binding runs were performed for PSD95-Ib and GRIP1-IIb consisting of 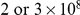 elementary MC steps.

### Order Parameters

The progress of binding is quantified using the two order parameters *Q*, the fraction of native inter-chain contacts, and 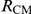, the distance between model and native peptide centers-of-mass (CM). To calculate *Q*, we determined initially a set of inter-chain amino acid contacts for each experimental domain-peptide structure. Two amino acids are considered in contact if any two non-H atoms, one from each amino acid, have a distance 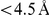. This yields sets of 28, 30, and 23 inter-chain native contacts for the domain-peptide structures 1BE9 (PSD-95), 1N7F (GRIP1), and 2PKU (PICK1), respectively. For PICK1-Ib, in the absence of an experimental ligand-bound structure for the 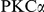 peptide, we use our minimum-energy conformation (see Figure 1F), which yields a set of 23 native contacts. In calculating *Q* for a peptide conformation, the fraction of native contacts formed is determined by applying the same contact definition. The CM distance is determined using 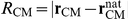, where 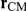 and 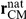 are the CMs of the model and native peptide conformations, respectively, calculated over the 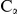 atoms of the last 4 amino acids.

## Supporting Information

Figure S1
**Example of a simulation trajectory.** One of the 5 Simulated Tempering runs performed for PSD95-If. The index *k* represents different temperatures chosen according to 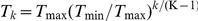, where 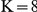 is the number of temperatures, 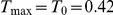, and 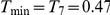. Changes in *k* are performed as ordinary MC updates. The figure shows, as functions of the number of MC steps, 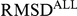, the total energy *E*, and the temperature index *k*.(TIFF)

Figure S2
**Conformational behavior of isolated peptide chains.** The radius of gyration, 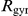, as a function of the temperature, *T*, for two different peptide sequences in isolation. 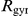 is calculated over all peptide 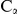 atoms. The relative variation in 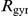 is around 2–3% for both sequences over the *T*s studied.(TIFF)

Figure S3
**Structural diversity of bound peptide conformations.** Superposition of a set of model peptide conformations (dark blue) with 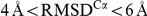 for PSD95-Ib (left) and GRIP1-IIb (right). The corresponding experimental domain-peptide complexes are shown in light blue (domain) and green (peptide).(TIFF)
