# Supplementary figures and images for: Binding Free Energy Landscape of Domain-Peptide Interactions
Source: PLoS Comput Biol. 2011 Aug 18;7(8):e1002131. doi: 10.1371/journal.pcbi.1002131 (PMC3158039; doi:10.1371/journal.pcbi.1002131)

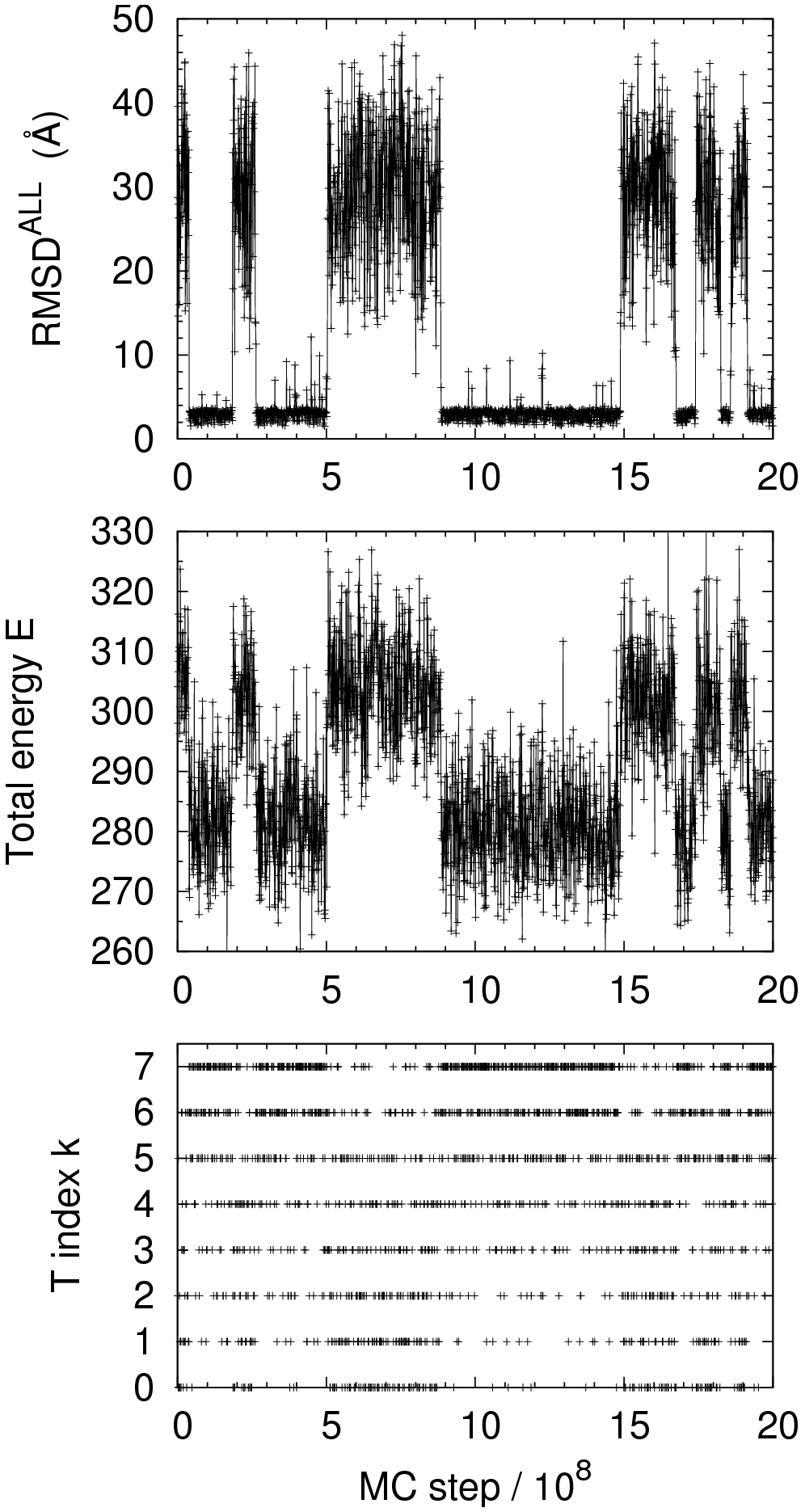

Supplement: Figure S1 — Example of a simulation trajectory. One of the 5 Simulated Tempering runs performed for PSD95-If. The index k represents different temperatures chosen according to , where is the number of temperatures, , and . Changes in k are performed as ordinary MC updates. The figure shows, as functions of the number of MC steps, , the total energy E, and the temperature index k. (TIFF) [file pcbi.1002131.s001.tif]

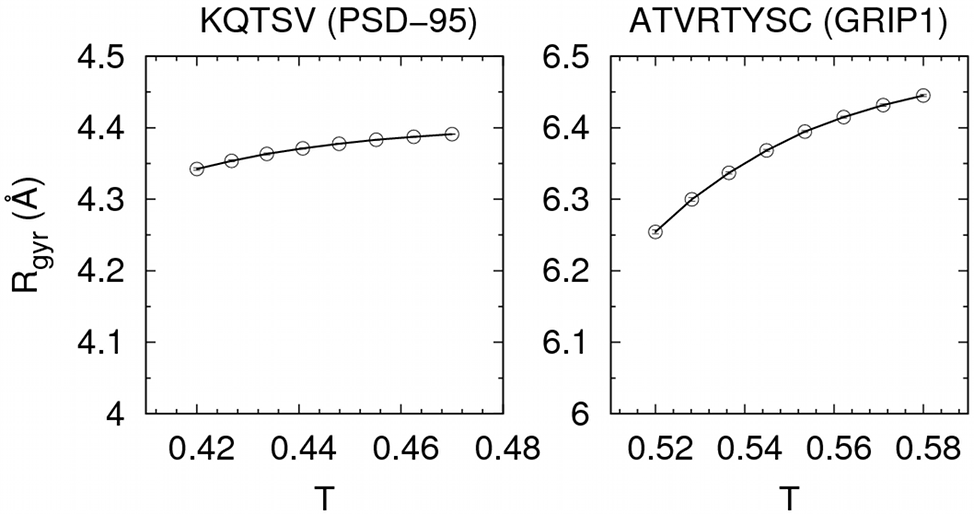

Supplement: Figure S2 — Conformational behavior of isolated peptide chains. The radius of gyration, , as a function of the temperature, T, for two different peptide sequences in isolation. is calculated over all peptide atoms. The relative variation in is around 2–3% for both sequences over the Ts studied. (TIFF) [file pcbi.1002131.s002.tiff]

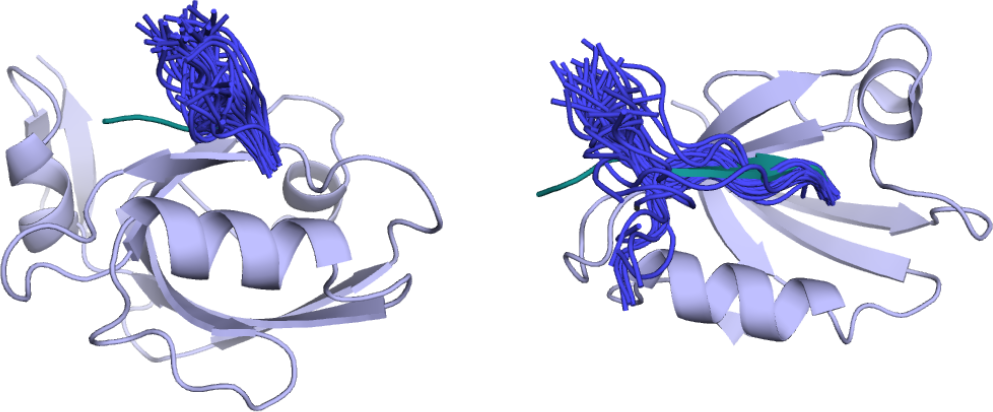

Supplement: Figure S3 — Structural diversity of bound peptide conformations. Superposition of a set of model peptide conformations (dark blue) with for PSD95-Ib (left) and GRIP1-IIb (right). The corresponding experimental domain-peptide complexes are shown in light blue (domain) and green (peptide). (TIFF) [file pcbi.1002131.s003.tiff]
